# Colossal Nernst power factor in topological semimetal NbSb_2_

**DOI:** 10.1038/s41467-022-35289-z

**Published:** 2022-12-09

**Authors:** Peng Li, Pengfei Qiu, Qing Xu, Jun Luo, Yifei Xiong, Jie Xiao, Niraj Aryal, Qiang Li, Lidong Chen, Xun Shi

**Affiliations:** 1 State Key Laboratory of High Performance Ceramics and Superfine Microstructure, Shanghai Institute of Ceramics, Chinese Academy of Sciences, 200050 Shanghai, China; 2 Center of Materials Science and Optoelectronics Engineering, University of Chinese Academy of Sciences, 100049 Beijing, China; 3 Key Laboratory of Infrared Imaging Materials and Devices, Shanghai Institute of Technical Physics, Chinese Academy of Sciences, 200083 Shanghai, China; 4 Condensed Matter Physics and Materials Science Division, Brookhaven National Laboratory, Upton, NY 11973-5000 USA; 5 Department of Physics and Astronomy, Stony Brook University, Stony Brook, NY 11794−3800 USA

**Keywords:** Thermoelectric devices and materials, Thermoelectrics

## Abstract

Today solid-state cooling technologies below liquid nitrogen boiling temperature (77 K), crucial to quantum information technology and probing quantum state of matter, are greatly limited due to the lack of good thermoelectric and/or thermomagnetic materials. Here, we report the discovery of colossal Nernst power factor of 3800 × 10^−4 ^W m^−1^ K^−2^ under 5 T at 25 K and high Nernst figure-of-merit of 71 × 10^−4 ^K^−1^ under 5 T at 20 K in topological semimetal NbSb_2_ single crystals. The observed high thermomagnetic performance is attributed to large Nernst thermopower and longitudinal electrical conductivity, and relatively low transverse thermal conductivity. The large and unsaturated Nernst thermopower is the result of the combination of highly desirable electronic structures of NbSb_2_ having compensated high mobility electrons and holes near Fermi level and strong phonon-drag effect. This discovery opens an avenue for exploring material option for the solid-state heat pumping below liquid nitrogen temperature.

## Abstract (web summary)

Today solid-state cooling technologies below 77 K are greatly limited. Here, the authors report the colossal Nernst power factor in NbSb_2_ single crystal, providing a high performance thermomagnetic material for solid-state cooling at low temperature.

## Introduction

Capable of converting heat into electricity and vice versa without moving parts and greenhouse emission, thermoelectricity plays an important role in solid-state energy harvesting and cooling^1–4^. Current thermoelectric (TE) technologies are largely developed for applications around room temperature (cooling/heating) and above (e.g., waste heat recovery), primarily due to the state-of-the-art TE materials exhibiting large electronic entropy, or a large TE power factor—a measurement of entropy transfer capability^5^. However, there is a demand now for TE applications at low temperatures, especially near or below liquid helium boiling point (4.2 K) heightened by applications in exploring the quantum state of matters^6^, quantum information science and technologies^7^, and space science and technologies^8^, among others.

**Fig. 1:**
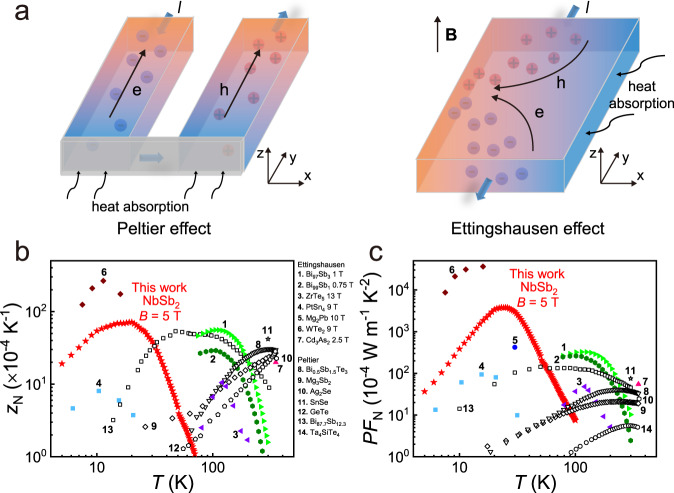
Peltier effect and Ettingshausen effect. **a** Schematic diagrams of the Peltier effect and Ettingshausen effect. Comparison of **b** the Nernst figure-of-merit (*z*_N_) and **c** Nernst power factor (*PF*_N_) for single-crystalline NbSb_2_ and other thermomagnetic materials^16,17,21,22,26,27,50^. The Peltier figure-of-merit (*z*) and Peltier power factor (*PF*) of typical TE materials are also included^34,38,40,41,47,48^.

TE refrigeration used today is based on the Peltier effect that has the advantages of accurate and fast temperature control, and nearly maintenance-free. As shown in Fig. 1a, when a longitudinal current flows through a thermopile, a longitudinal temperature gradient is formed, yielding the reduction of temperature at one end of the thermopile. Vast majority of TE devices used today have longitudinal configurations (Fig. 1a) made of n- and p-type elements connected in series where electrical and thermal resistive contact are primary sources of reduced efficiency^9–12^. A maximum temperature drop of around 70 K has been achieved in typical Bi_2_Te_3_-based TE devices at room temperature by the Peltier effect^4^. However, such technology is greatly limited at temperatures below liquid nitrogen boiling point (~77 K) due to the lack of high-performance TE materials in the low-temperature range (Fig. 1b, c). The physical reason behind this phenomenon is understood as follows. It has been argued that a good conventional TE semiconductor usually has a bandgap ~10 *k*_B_*T* (where *k*_B_ is the Boltzmann constant and *T* is the working temperature)^13^. In order to have it efficiently work below 77 K, the bandgap should be less than 66 meV. This leads to the great difficulty of finding the potential high-performance TE materials for Peltier refrigeration in the low-temperature range since semiconductors with bandgap less than 66 meV are rare.

Ettingshausen refrigerator is a transverse TE device that provides cooling orthogonal to the applied voltage that greatly simplifies a thermopile structure, in which only the electrical contact is required at the colder side of the thermoelectric material that does not require compatible p- and n-type elements, and can reduce the thermal resistance. The Ettingshausen effect is shown in Fig. 1a. When a longitudinal current (along the *y* direction) flows through a thermomagnetic material under magnetic field, a transverse temperature gradient (along the *x* direction) will be formed, yielding the reduction of temperature at the material’s transverse side surface. The cooling efficiency of Ettingshausen refrigeration is determined by the material’s electrical and thermal transport properties in orthogonal directions, which can be evaluated by a comprehensive parameter named as the Nernst figure-of-merit^14^, ${z}_{{{{{{\rm{N}}}}}}}=\frac{{S}_{{yx}}^{2}{\sigma }_{{yy}}}{{\kappa }_{{xx}}}$, where *S*_*yx*_ is the Nernst thermopower, *σ*_*yy*_ is the longitudinal electrical conductivity, *κ*_*xx*_ is the transverse thermal conductivity, and magnetic field is along the *z* direction, respectively. In contrast to the longitudinal TE power factor *PF* ($={S}^{2}\sigma$, where *S* and *σ* are the Seebeck thermopower and electrical conductivity along the same direction, respectively), the Nernst power factor ${{PF}}_{{{{{{\rm{N}}}}}}}={S}_{{yx}}^{2}{\sigma }_{{yy}}$ is used to determine the transverse pumping power. Electrons and holes moving in the opposite direction driven by the longitudinal current, can carry both charge and energy in the same transverse direction synergistically under magnetic field, resulting in a doubling of the transverse temperature gradient. Therefore, semimetals with zero bandgap or slight band overlap are particularly suitable for the Ettingshausen cooling at low temperatures below 77 K.

Although the Ettingshausen effect was discovered in 1886^15^, Ettingshausen refrigeration has progressed far less than Peltier refrigeration. For a long time, the investigation is only limited in a few thermomagnetic materials, such as Bi–Sb alloys^16,17^ and In-Sb alloys^18^. The peak *z*_N_ values of single-crystalline Bi_97_Sb_3_^16^ and Bi_99_Sb_1_^17^ are 55 × 10^−4 ^K^−1^ under 1 T and 29 × 10^−4 ^K^−1^ under 0.75 T at 115 K, respectively (Fig. 1b). Recently, the discovery of topological semimetals with high carrier mobility has rejuvenated the investigation of Ettingshausen effect^19–27^. It is noted that the Dirac-like linear electronic band dispersion near Fermi level in topological semimetals^28^ can lead to an energy-independent electronic density of states that increases linearly with magnetic field, thus create huge electronic entropy^20,29^. Indeed, the peak *z*_N_ of Dirac semimetal ZrTe_5_ was reported to reach 10.5 × 10^−4 ^K^−1^ under 13 T at 120 K^21^. Nodal-line semimetal PtSn_4_ has a peak *z*_N_ of 8 × 10^−4 ^ K^−1^ under 9 T at 10 K^22^. Most recently, Pan et al. reported an ultrahigh *z*_N_ of 265 × 10^−4 ^ K^−1^ under 9 T at 11.3 K in single-crystalline Weyl semimetal WTe_2_^27^. This value is already much higher than that of Bi–Sb alloys (Fig. 1b), which was recently shown to be also a topological semimetal in specific chemical composition range after all^30^. These results motivate the discovery of new thermomagnetic materials with high *z*_N_ below liquid nitrogen temperature from topological semimetals.

In this work, we report that topological semimetal NbSb_2_ single crystal is a promising high-performance thermomagnetic material with a colossal *PF*_N_ of 3800 × 10^−4 ^W m^−1^ K^−2^ under 5 T at 25 K (Fig. 1c) and a high *z*_N_ of 71 × 10^−4 ^ K^−1^ under 5 T at 20 K (Fig. 1b), much higher than most TE and thermomagnetic materials below 77 K. We found that the performance in NbSb_2_ benefits from the combination of nearly identical electron and hole concentrations, high electron/hole carrier mobilities, and additional phonon-drag effect.

## Results

### Crystal structure

**Fig. 2:**
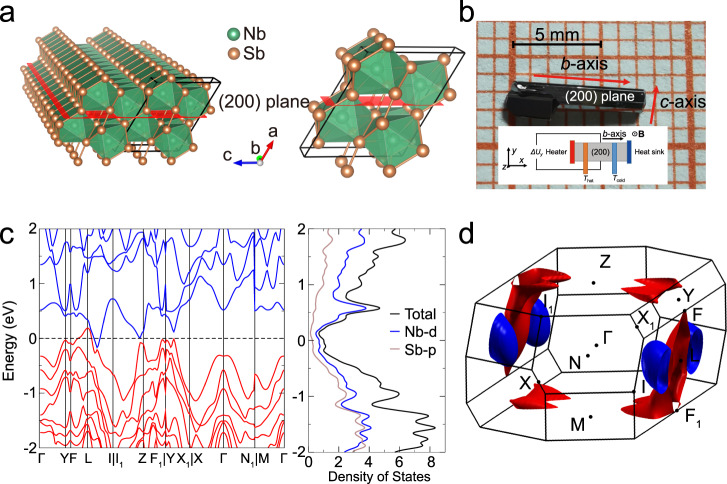
Crystal structure and band structure of NbSb_2_. **a** Crystal structure of NbSb_2_ from different perspectives. **b** Optical image of NbSb_2_ single crystal grown in this work. The inset shows the measurement direction of the Nernst thermopower. **c** Calculated band structure, density of states, and **d** Fermi surface with the spin–orbit coupling (SOC) for NbSb_2_. The red and blue pockets denote the hole and electron pockets, respectively.

NbSb_2_ is a topological semimetal^31^. It crystallizes in centrosymmetric monoclinic structure with the space group of ${C}_{2/m}$. The schematics of its crystal structure is shown in Fig. 2a. The Nb atom is enclosed in a hendecahedron composed of Sb atoms. The hendecahedrons are connected with each other in the way of face-to-face along the *b* axis and edge-to-edge along the *c* axis, forming an atomic layer parallel to *bc* plane. The lattice parameters for NbSb_2_ are *a* = 10.239 Å, *b* = 3.632 Å, *c* = 8.333 Å, and *β* = 120.07°^32^. Figure 2b shows the NbSb_2_ single crystal grown by the chemical vapor transport method. The NbSb_2_ single crystal has a bar-like shape with the length about 7 mm and the width about 1–2 mm. The X-ray characterization performed on the upper surface (Supplementary Fig. 1a) shows that strong (200), (400), and (600) diffraction peaks are observed, indicating the high quality of our NbSb_2_ single crystal. Supplementary Fig. 1b shows that Nb and Sb are homogeneously distributed inside the matrix, consistent with the pure phase detected by XRD measurement.

### Band structure

Figure 2c shows the calculated band structure of NbSb_2_ with the inclusion of spin–orbit coupling (SOC) effect. The Fermi level crosses the conduction band on the path from *L* to *I* and the valence band near *L*, rendering it a typical semimetal. The energy overlap between conduction band and valence band is about 350 meV. From the Fermi surface (FS) plotted in Fig. 2d, we can identify one electron pocket (blue shell) and one hole pocket (red shell) in the first Brillouin zone. The calculated FS area on the *ab* plane is comparable to the experimentally measured area from the quantum oscillation measurement^33^. A plot showing variation of the calculated FS area with chemical potential and comparison with the experimental value is shown in Supplementary Fig. 2, with the details shown in Supplementary Note 1. The similarity between the calculated and measured FS areas provides validity to the density functional theory (DFT)-predicted electronic structure. The electron pocket and the hole pocket have nearly the same volume leading to well-compensated electrons and holes near the Fermi level. Under orthogonal applied magnetic field and current, the electrons and holes in these pockets moving in the opposite direction along the longitudinal current are deflected in the same transverse direction, which can strengthen the Ettingshausen effect.

### Transport properties

Supplementary Fig. 3a, b shows the temperature dependences of adiabatic transverse electrical resistivity *ρ*_*xx*_ and Hall resistivity *ρ*_*yx*_ of single-crystalline NbSb_2_ under different magnetic fields *B*. When *B* = 0, the *ρ*_*xx*_ rises with increasing temperature, showing typical metal-like conduction behavior. The *ρ*_*xx*_ is ~2 × 10^−9^ Ω m at 5 K, which is about 3–4 orders of magnitude lower than those of typical TE materials for Peltier refrigeration^4,34^. Upon applying magnetic field, the *ρ*_*xx*_ at temperatures below 100 K is greatly increased, a characteristic feature of topological semimetals^28,35^. The magnetoresistance (MR) ratio of single-crystalline NbSb_2_ under 9 T at 5 K is 1.3 × 10^5^%, comparable to those of extremely large magnetoresistance (XMR) materials reported before, such as MR = 8.5 × 10^5^% for NbP under 9 T at 1.85 K^36^, 4.5 × 10^5^% for WTe_2_ under 14.7 T at 4.5 K^35^, and 5 × 10^5^% for PtSn_4_ under 14 T at 1.8 K^37^. Supplementary Fig. 3b shows that the absolute value of Hall resistivity (|*ρ*_*yx*_|) firstly decreases with increasing temperature, reaches a minimum at about 100 K, and then increases at a higher temperature. Under the same magnetic field, the |*ρ*_*yx*_| is much lower than the *ρ*_*xx*_.

**Fig. 3:**
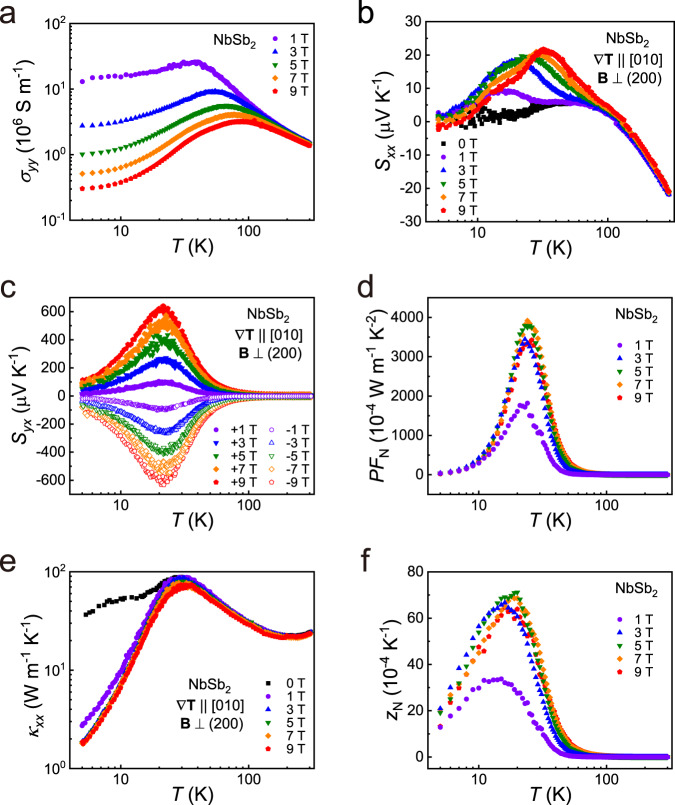
Electrical and thermal properties of NbSb_2_ for Ettingshausen refrigeration. Temperature dependences of **a** electrical conductivity (*σ*_*yy*_), **b** Seebeck thermopower (*S*_*xx*_), **c** Nernst thermopower (*S*_*yx*_), **d** Nernst power factor (*PF*_N_), **e** transverse thermal conductivity (*κ*_*xx*_), and **f** Nernst figure-of-merit (*z*_N_) of single-crystalline NbSb_2_ under different magnetic fields and adiabatic condition. In thermal transport measurements, the temperature gradient $\nabla$**T** is parallel to the [010] direction and the magnetic field **B** is perpendicular to the (200) plane.

To evaluate the Nernst figure-of-merit, we need to know the longitudinal conductivity *σ*_*yy*_, which can be calculated by the equation

1 $${\sigma }_{{yy}}=\frac{{\rho }_{{xx}}}{{\rho }_{{xx}}{\rho }_{{yy}}-{\rho }_{{yx}}{\rho }_{{xy}}}=\frac{{\rho }_{{xx}}}{{({\rho }_{{yy}}/{\rho }_{{xx}})\rho }_{{xx}}^{2}+{\rho }_{{yx}}^{2}}$$

where *ρ*_*yy*_ is the longitudinal electrical resistivity. The value of *ρ*_*yy*_/*ρ*_*xx*_ is determined by measuring the electrical resistivity along the *b* axis (*ρ*_*xx*_) and the electrical resistivity along the *c* axis (*ρ*_*yy*_) of a thin square single-crystalline NbSb_2_ sample (Supplementary Figs. 4a, b). It seems that the electrical resistivities behavior of NbSb_2_ is more anisotropic at low temperatures, but less anisotropic at room temperature. Under the assumption that −*ρ*_*xy*_ is equal to *ρ*_*yx*_, the *σ*_*yy*_ under different magnetic fields is calculated and shown in Fig. 3a. The *σ*_*yy*_ first increases with increasing temperature, reaches a maximum, and then decreases with further increasing temperature. The temperature corresponding to the maximum *σ*_*yy*_ is gradually shifted from 35 K under *B* = 1 T to 85 K under *B* = 9 T.

The adiabatic Seebeck thermopower *S*_*xx*_ below 100 K is very small under *B* = 0 T (Fig. 3b), with the absolute value |*S*_*xx*_ | less than 5 μV K^−1^. Below 100 K, it increases modestly with increasing magnetic field, with the peak value around 20 μV K^−1^ even under *B* = 9 T. Above 100 K, the |*S*_*xx*_ | increases with increasing temperature, but the maximum is still much lower than those of conventional TE materials^34,38–40^. Such low *S*_*xx*_ values are consistent with the semimetal feature of NbSb_2_ (Fig. 2c).

Figure 3c shows the temperature dependence of adiabatic Nernst thermopower *S*_*yx*_ of single-crystalline NbSb_2_. Under a magnetic field, the absolute value of *S*_*yx*_ initially increases with increasing temperature, reaches the maximum value of around 21 K, and then decreases at higher temperatures. Similar behavior is observed when the direction of the magnetic field is reversed, with the sign of *S*_*yx*_ is reversed accordingly. The maximum *S*_*yx*_ is about 616 μV K^−1^ under 9 T at 21 K, about 30 times of the maximum *S*_*xx*_. Likewise, as shown in Supplementary Note 2, the thermal Hall effect has little influence on the *S*_*yx*_ measurement.

The adiabatic Nernst power factor *PF*_N_ ($={S}_{{yx}}^{2}{\sigma }_{{yy}}$) of single-crystalline NbSb_2_ under different magnetic fields is shown in Fig. 3d. The *PF*_N_ firstly increases with increasing temperature, reaches a peak around 25 K, and then decreases at higher temperatures. At $B$ = 1 T, the *PF*_N_ reaches 1750 × 10^−4 ^W m^−1^ K^−2^ at 25 K. As shown in Fig. 1c, this value is already much higher than the best Peltier *PF* of the TE materials, such as 41 × 10^−4 ^W m^−1^ K^−2^ for Bi_2_Te_3_^34^, 75 × 10^−4 ^W m^−1^ K^−2^ for SnSe^41^, and 25 × 10^−4 ^W m^−1^ K^−2^ for Mg_3_Sb_2_^34^. This result is very encouraging as many permanent magnets can easily provide 1 T magnetic field, thus utilizing single-crystalline NbSb_2_ for the Ettingshausen cooling is practically viable. At *B* = 5 T, the *PF*_N_ is further enhanced to 3800 × 10^−4 ^W m^−1^ K^−2^ at 25 K. As shown in Fig. 1c, this value is much higher than those of single-crystalline PtSn_4_^22^ and single-crystalline Mg_2_Pb^26^. It is only lower than that for WTe_2_, which has the *PF*_N_ up to 36,000 × 10^−4 ^ W m^−1^ K^−2^ under 9 T at 15.9 K^27^.

Figure 3e shows the adiabatic transverse thermal conductivity *κ*_*xx*_ of single-crystalline NbSb_2_ from 5 to 300 K measured by using the four-probe method. At *B* = 0, the *κ*_*xx*_ increases with increasing temperature, reaches a peak of 90 W m^−1^ K^−1^ around 30 K, and then decreases with further increasing temperature. At 300 K, the *κ*_*xx*_ is around 24 W m^−1^ K^−1^, which is much higher than those of the TE materials for Peiter refrigeration, such as 1.1 W m^−1^ K^−1^ for Bi_2_Te_3_^34^, 3.0 W m^−1^ K^−1^ for filled skutterudites^42^, and 1.0 W m^−1^ K^−1^ for Cu_2_Se^39^. However, it is noteworthy that the peak *κ*_*xx*_ of single-crystalline NbSb_2_ is lower than those of many thermomagnetic materials for Ettingshausen refrigeration, such as 1290 W m^−1^ K^−1^ for single-crystalline NbP under 8 T^43^, 1586 W m^−1^ K^−1^ for single-crystalline TaP under 9 T^20^, and 215 W m^−1^ K^−1^ for single-crystalline WTe_2_ under 9 T^27^. When the magnetic field is applied, the *κ*_*xx*_ of single-crystalline NbSb_2_ at low temperatures is significantly decreased. As shown in Supplementary Fig. 5, the *κ*_*xx*_ at 5 K is 35.9 W m^−1^ K^−1^ when *B* = 0 T, but only 2.7 W m^−1^ K^−1^ when *B* = 1 T. When the magnetic field is increased to 3 T, the *κ*_*xx*_ is further decreased. However, under a higher magnetic field, the *κ*_*xx*_ is almost unchanged. Such *κ*_*xx*_ reduction under magnetic field is caused by the suppression of the contribution of carriers in thermal transports. Moreover, as shown in Supplementary Fig. 6, the estimated isothermal *κ*_*xx*_ is slightly smaller than the measured adiabatic *κ*_*xx*_.

The measured *κ*_*xx*_ in Fig. 3e is mainly composed of the lattice thermal conductivity *κ*_l_ and carrier thermal conductivity *κ*_e_. Under magnetic field, their relationship can be expressed by the empirical formula^20,22,44^

2 $${\kappa }_{{xx}}\left(B,T\right)={\kappa }_{{{{{{\rm{l}}}}}}}\left(T\right)+{\kappa }_{{{{{{\rm{e}}}}}}}\left(B,T\right)={\kappa }_{{{{{{\rm{l}}}}}}}\left(T\right)+\frac{{\kappa }_{{{{{{\rm{e}}}}}}}\left(0,\,T\right)}{1+\eta {B}^{s}}$$

where *η* and *s* are the two factors related to the thermal mobility and scattering mechanism, respectively. The increase of *B* will suppress the contribution of carriers, which is responsible for the reduction of *κ*_*xx*_ under high magnetic field (Fig. 3e). By using Eq. (2), the measured *κ*_*xx*_ data of NbSb_2_ under different *B* and *T* are fitted. The fitting results are shown in Supplementary Fig. 5a and Supplementary Table 1. The *κ*_l_ increases with increasing temperature, reaching the maximum around 25 K, and then decreases at a higher temperature. The maximum is caused by the transition from the *κ*_l_ ~*T*^3^ dependence at low temperature to *κ*_l_ ~*T*^−1^ dependence at high temperature^45^. Based on the fitted *κ*_e_, the Lorenz number *L* can be calculated from the Wiedemann–Franz law. As shown in Supplementary Fig. 5b, the *L* at low temperatures is significantly lower than the Sommerfeld value *L*_0_ = 2.44 × 10^−8 ^W Ω K^−2^, indicating the violation of Wiedemann–Franz law. The ratio of the Lorenz number to Sommerfeld value (*L*/*L*_0_) decreases from around 1 near room temperature to the minimum value of 0.29 at *T* = 15 K, and then increases at a lower temperature, reaching 0.59 at 5 K. This trend is similar to the phenomenon found in WP_2_ by Jaoui et al.^46^. The violation of WF law might be caused by the inelastic scattering of carriers, while the upturn of *L*/*L*_0_ below 15 K might be caused by the changed carrier scattering mechanism from the inelastic scattering into the elastic scattering from the impurities.

The adiabatic Nernst figure-of-merit *z*_N_ ($=\frac{{S}_{{yx}}^{2}{\sigma }_{{yy}}}{{\kappa }_{{xx}}}$) of single-crystalline NbSb_2_ under different magnetic fields is shown in Fig. 3f. The corresponding adiabatic *z*_N_*T* are shown in Supplementary Fig. 7a. The *z*_N_ and *z*_N_*T* increase with increasing temperature, reach the peak value around 20 K, and then decrease at a higher temperature. Due to the enhanced *PF*_N_ and the reduced *κ*_*xx*_, the *z*_N_ of single-crystalline NbSb_2_ is greatly enhanced by magnetic field. A maximum of *z*_N_ is 33 × 10^−4 ^K^−1^ under 1 T at 15 K, which is about six times that of PtSn_4_ under 9 T at 15 K^22^ (Fig. 1b). The *z*_N_ is further enhanced to 71 × 10^−4 ^K^−1^ under 5 T at 20 K, corresponding to the adiabatic *z*_N_*T* of 0.14 and the isothermal *z*_N_*T* of 0.16 (Supplementary Fig. 6b). With further increasing the magnetic field, the *z*_N_ and *z*_N_*T* tend to saturate (Supplementary Fig. 7b and Supplementary Fig. 7c). As shown in Fig. 1b, the *z*_N_ of single-crystalline NbSb_2_ is higher than the Peiter figure-of-merit *z* of all the TE materials^34,38,40,41,47,48^. It is among the best thermomagnetic materials for Ettingshausen refrigeration reported so far. More importantly, the high *z*_N_ and *z*_N_*T* of NbSb_2_ appear in the temperature range of 5–30 K (Fig. 1b and Supplementary Fig. 7d), which can well satisfy the requirement of refrigeration below liquid nitrogen temperature.

### Potential application

Based on the measured thermomagnetic properties, the maximum temperature difference (Δ*T*_max_) and the maximum specific heat pumping power (*P*_max_) of the present single-crystal NbSb_2_ can be estimated by the following equations^14,26^

3 $$\triangle {T}_{{{\max }}}=\frac{1}{2}{z}_{{{{{{\rm{N}}}}}}}^{{{{{{\rm{iso}}}}}}}{T}_{{{{{{\rm{c}}}}}}}^{2}$$

4 $${P}_{{{\max }}}=\frac{{S}_{{yx}}^{2}{T}_{{{{{{\rm{c}}}}}}}^{2}{\sigma }_{{yy}}A}{2{lm}}=\frac{{S}_{{yx}}^{2}{T}_{{{{{{\rm{c}}}}}}}^{2}{\sigma }_{{yy}}}{2D{l}^{2}}$$

where ${z}_{{{{{{\rm{N}}}}}}}^{{{{{{\rm{iso}}}}}}}$ is the isothermal figure-of-merit, *T*_c_ is the cold-end temperature, *l* and *A* are the thickness and cross-sectional area of a cuboid sample along the direction of heat flow, *m* and *D* are the mass and density of the sample, respectively. Under *B* = 5 T and *T*_c_ = 25 K, the Δ*T*_max_ of NbSb_2_ single crystal is about 2.0 K. Particularly, in the condition of *B* = 5 T and *T*_c_ = 25 K, the theoretical *P*_max_ of a cuboid sample with *l* = 1 mm is about 14.2 W g^−1^, which is much higher than the compression refrigerator with gas refrigerants^26^ (e.g., *P*_max_ = 0.05 W g^−1^ for He at 5 K, 0.1 W g^−1^ for H_2_ at 26 K, and 1.0 W g^−1^ for N_2_ at 93 K). Furthermore, the mechanical workability of NbSb_2_ single crystal is very good. As shown in Supplementary Fig. 8, it can be easily machined into regular thin square and rectangle without cracking. This can facilitate the fabrication of the classic exponential shape for Ettingshausen refrigeration^49^.

### Two-carrier model

**Fig. 4:**
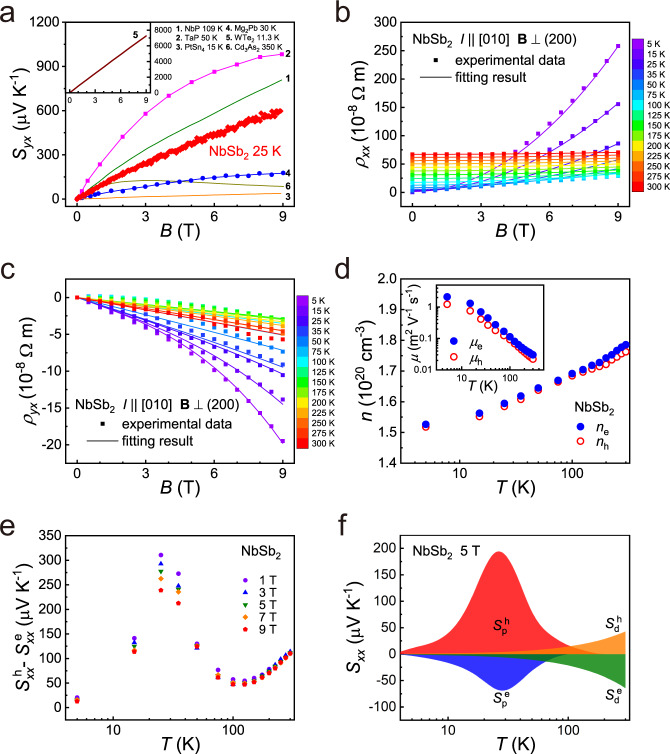
Detailed electrical transports for NbSb_2_ single crystal. **a** Nernst thermopower (*S*_*yx*_) of single-crystalline NbSb_2_ as a function of magnetic field *B* at 25 K. The data for PtSn_4_^22^, Cd_3_As_2_^50^, Mg_2_Pb^26^, NbP^19^, TaP^20^, and WTe_2_^27^ are included for comparison. **b** Fitting of the transverse resistivity *ρ*_*xx*_(*B*) and **c** Hall resistivity *ρ*_*yx*_(*B*) of single-crystalline NbSb_2_ under different temperatures. The symbols are experimental data and the lines are the fitting curves. In electrical transport measurements, the current *I* is parallel to the [010] direction and the magnetic field **B** is perpendicular to the (200) plane. **d** Carrier concentrations (*n*_e_ and *n*_h_) and carrier mobilities (*μ*_e_ and *μ*_h_) of single-crystalline NbSb_2_. **e** Temperature dependence of the difference between Seebeck thermopower of electrons and holes (${S}_{{xx}}^{{{{{{\rm{h}}}}}}}-{S}_{{xx}}^{{{{{{\rm{e}}}}}}}$) of single-crystalline NbSb_2_ under different magnetic fields. **f** Seebeck thermopower of electrons and holes related to the charge carrier diffusion processes (${S}_{{{{{{\rm{d}}}}}}}^{{{{{{\rm{e}}}}}}}$ and ${S}_{{{{{{\rm{d}}}}}}}^{{{{{{\rm{h}}}}}}}$) and phonons (${S}_{{{{{{\rm{p}}}}}}}^{{{{{{\rm{e}}}}}}}$ and ${S}_{{{{{{\rm{p}}}}}}}^{{{{{{\rm{h}}}}}}}$) at 5 T, respectively.

The large and unsaturated *S*_*yx*_ under a high magnetic field is indispensable for realizing high *PF*_N_ and *z*_N_ of thermomagnetic materials. As shown in Fig. 4a, beyond the present NbSb_2_, nearly all the reported good thermomagnetic materials possess such character^19,20,22,26,27,50^. NbSb_2_ is a semimetal with the Fermi level simultaneously crossing the conduction band and valence band (Fig. 2c). Thus, both electrons and holes will take part in the electrical transports. By using Supplementary Eqs. (13) and (14), the electron (or hole) carrier concentration *n*_e_ (or *n*_h_), and electron (or hole) carrier mobility *μ*_e_ (or *μ*_h_) in NbSb_2_ can be obtained by fitting the two-carrier model to the measured transverse resistivity *ρ*_*xx*_(*B*) and Hall resistivity *ρ*_*yx*_(*B*). This model can well fit the *ρ*_*xx*_(*B*) and *ρ*_*yx*_(*B*) data over 5–300 K (Fig. 4b, c). The *n*_e_ and *n*_h_ are almost the same with each other ~10^20 ^cm^−3^ over the entire temperature. Likewise, the inset in Fig. 4d shows that the *μ*_e_ and *μ*_h_ of single-crystalline NbSb_2_ are also comparable over the entire temperature range. In a two-carrier model^51^ with constant relaxation time approximation and under the ideal conditions of *n*_e_ = *n*_h_ and *μ*_e_ = *μ*_h_ = $\bar{\mu }$, the *S*_*yx*_ can be expressed as

5 $${S}_{{yx}}=\frac{\bar{\mu }B}{2}\left({S}_{{xx}}^{{{{{{\rm{h}}}}}}}-{S}_{{xx}}^{{{{{{\rm{e}}}}}}}\right)$$

where ${S}_{{xx}}^{{{{{{\rm{e}}}}}}}$ and ${S}_{{xx}}^{{{{{{\rm{h}}}}}}}$ are the Seebeck thermopower of electrons and holes under the magnetic field *B*, respectively. The details about how Eq. (5) is obtained can be found in Supplementary Note 3. Different from the one-carrier model in which a saturated *S*_*yx*_ is observed under large magnetic field, the two-carrier model based on Eq. (5) gives an unsaturated *S*_*yx*_ when magnetic field increases, this is consistent with the measured *S*_*yx*_ vs. *B* behavior of single-crystalline NbSb_2_ shown in Fig. 4a.

The inset in Fig. 4d shows that the *μ*_e_ and *μ*_h_ of single-crystalline NbSb_2_ are very large at low temperature, reaching *μ*_e_ = 2.1 m^2^ V^−1^ s^−1^ and *μ*_*h*_ = 1.2 m^2^ V^−1^ s^−1^ at 5 K. These values are comparable with the high mobility found in the extremely large magnetoresistance materials, such as Cd_3_As_2_ (*μ*_e_ = 6.5 m^2^ V^−1^ s^−1^ and *μ*_h_ = 0.5 m^2^ V^−1^ s^−1^ at 10 K)^50^, PtSn_4_ (*μ*_e_ = 7.6 m^2^ V^−1^ s^−1^ and *μ*_h_ = 7.6 m^2^ V^−1^ s^−1^ at 2 K)^22^, LaBi (*μ*_e_ = 2.6 m^2^ V^−1^ s^−1^ and *μ*_h_ = 3.3 m^2^ V^−1^ s^−1^ at 2 K)^52^. The observed high *μ*_e_ and *μ*_h_ are also consistent with the Dirac-like band dispersion of NbSb_2_ near the Fermi level (Fig. 2c). The large *μ*_e_ and *μ*_h_ are one important reason for the large *S*_*yx*_ of single-crystalline NbSb_2_.

In addition, it is instructive to plot $({S}_{{xx}}^{{{{{{\rm{h}}}}}}}-{S}_{{xx}}^{{{{{{\rm{e}}}}}}})$ of single-crystalline NbSb_2_ under different temperatures and magnetic fields. In Fig. 4e, $({S}_{{xx}}^{{{{{{\rm{h}}}}}}}-{S}_{{xx}}^{{{{{{\rm{e}}}}}}})$ shows a local peak at 25 K, which is believed to have a consequence for the observed colossal Nernst power factor. In thermoelectrics, such extra-large thermopower peak at low temperature is usually caused by the phonon-drag effect^14,51^. With increasing temperature, the phonons with higher momentum are excited. When the momentum of the long-wave acoustic phonons is similar with that of the carriers on the Fermi surface, the phonon-drag effect occurs, leading to the appearance of a peak in the Seebeck thermopower curve at low temperature. The Seebeck thermopower of a material can be written as *S*_*xx*_ = *S*_d_ +*S*_p_, where *S*_d_ is related to the charge carrier diffusion processes and *S*_p_ is related to phonons. In a degenerate limit, the *S*_d_ usually has linear temperature dependence^53^. The estimation details of ${S}_{{xx}}^{{{{{{\rm{e}}}}}}}$ and ${S}_{{xx}}^{{{{{{\rm{h}}}}}}}$ are shown in Supplementary Note 4. However, as presented in Supplementary Fig. 9, both ${S}_{{xx}}^{{{{{{\rm{e}}}}}}}$ and ${S}_{{xx}}^{{{{{{\rm{h}}}}}}}$ deviate off the linear temperature dependence below 100 K, indicating the non-negligible *S*_p_ in single-crystalline NbSb_2_ at low temperatures. By subtracting the *S*_d_ from the ${S}_{{xx}}^{{{{{{\rm{e}}}}}}}$ and ${S}_{{xx}}^{{{{{{\rm{h}}}}}}}$, the ${S}_{{{{{{\rm{p}}}}}}}^{{{{{{\rm{e}}}}}}}$ and ${S}_{{{{{{\rm{p}}}}}}}^{{{{{{\rm{h}}}}}}}$ can be estimated, with the details shown in Supplementary Note 5. As shown in Fig. 4f, the absolute values of ${S}_{{{{{{\rm{p}}}}}}}^{{{{{{\rm{e}}}}}}}$ and ${S}_{{{{{{\rm{p}}}}}}}^{{{{{{\rm{h}}}}}}}$ show the maxima value of 75 μV K^−1^ and 193 μV K^−1^ around 25 K, much larger than the ${S}_{{{{{{\rm{d}}}}}}}^{{{{{{\rm{e}}}}}}}$ (5.4 μV K^−1^) and ${S}_{{{{{{\rm{d}}}}}}}^{{{{{{\rm{h}}}}}}}$ (3.6 μV K^−1^) at the same temperature, respectively. Consequently, the synergistic effect of ${S}_{{{{{{\rm{p}}}}}}}^{{{{{{\rm{e}}}}}}}$ and ${S}_{{{{{{\rm{p}}}}}}}^{{{{{{\rm{h}}}}}}}$ greatly improves the total Ettingshausen effect in the single-crystalline NbSb_2_. At higher temperature, the phonon-drag effect is quickly weakened since the significantly excited high-frequency phonons lead to the reduction of the relaxation time of long-wave acoustic phonons^14^. Thus, the ${S}_{{{{{{\rm{p}}}}}}}^{{{{{{\rm{e}}}}}}}$ and ${S}_{{{{{{\rm{p}}}}}}}^{{{{{{\rm{h}}}}}}}$ are quickly decreased after reaching the maxima values. Above 125 K, the electrical transports are mainly determined by the carrier diffusion process. At this time, the measured *S*_*yx*_ is comparable with the theoretical value of $283\bar{\mu }/{E}_{{{{{{\rm{F}}}}}}}T$ (Supplementary Fig. 10)^54^, where *E*_F_ = 1200 K is derived from the relation ${E}_{{{{{{\rm{F}}}}}}}=\frac{{\hslash }^{2}}{2m}{(3{\pi }^{2}n)}^{2/3}$ (see ref. 55), with the carrier concentration *n* equaling to 1.5 × 10^20 ^ cm^−3^ and *m* equaling to the free electron mass *m*_0_. These prove that the fitted *μ* and *n* in Fig. 4d are reasonable.

## Discussion

In summary, we report a colossal Nernst power factor of 3800 × 10^−4 ^W m^−1^ K^−2^ under 5 T at 25 K and a high Nernst figure-of-merit *z*_N_ with of 71 × 10^−4 ^K^−1^ under 5 T at 20 K in single-crystalline NbSb_2_. There are a number of factors synergistically contributed to the large and unsaturated Nernst thermopower *S*_*yx*_ under magnetic field: (1) a favorable band structure providing nearly identical electron and hole concentrations at Fermi level, (2) extraordinary high electron and hole mobilities benefiting from the Dirac-like dispersion of low energy excitations common to several well-known topological semimetals, and (3) strong phonon-drag effect. The phonon-drag effect derived from our data analysis suggests phonon can play an important role in the transport process of Dirac fermions, which is another interesting phenomenon worth of further investigation. This work provides a new material option for the solid-state heat pumping below liquid nitrogen temperature.

## Methods

### Sample synthesis

NbSb_2_ single crystal was synthesized by the chemical vapor transport method in two steps. First, the polycrystalline powder was synthesized by solid-state reaction. The niobium powder (alfa, 99.99%) and antimony shot (alfa, 99.9999%) with stoichiometry 1:2 was encapsulated in a vacuum quartz tube and reacted at 1023 K for 48 h. Next, the polycrystalline NbSb_2_ powders and 0.3 g iodine were sealed in another vacuum quartz tube. The quartz tube was placed in a horizontal furnace with a temperature gradient for 2 weeks. The hot end temperature and cold end temperature of the quartz tube are 1373 K and 1273 K, respectively. Finally, shiny and bar-like single crystals appear in the cold end of the quartz tube.

### Characterization and transport property measurements

The phase composition of the single-crystalline NbSb_2_ was characterized by X-ray diffraction (XRD, D/max-2550 V, Rigaku, Japan) and scanning electron microscopy (SEM, ZEISS supra-55, Germany) with energy-dispersive X-ray spectroscopy (EDS, Oxford, UK). The electrical and thermal transport properties of single-crystalline NbSb_2_ were measured under the magnetic field by using physical property measurement system (PPMS, Quantum design, USA). The alternating current was used in the electrical conductivity measurement with the purpose to eliminate the thermal Hall effect. The transverse resistivity and Hall resistivity were measured by the four-probe method and the five-probe method, respectively. The Seebeck thermopower was measured on a standard thermal transport option (TTO) platform. The Nernst thermopower was measured on a modified TTO platform, where the Cu wires for measuring voltage signals were separated from the Cernox 1050 thermometers. All measurements of thermal transport were performed by using the four-probe method. The details can be found in Supplementary Note 6 and Supplementary Fig. 11a, b. The measurement direction was marked in the inset of Fig. 2b, which was the same as that of the Seebeck thermopower. Taking *b* axis as the *x* direction and *c* axis as the *y* direction, the magnetic field was applied in the *z* direction perpendicular to the *bc* plane. In addition, via comparing with the thermal conductivity of the sample with and without adhering Cu wires (Supplementary Fig. 12), it is concluded that the Cu wires have little influence on the measurement.

### Calculation

First-principles calculations were carried out using Quantum espresso software package^56^ with the lattice parameters given in the materials project^57^. Perdew–Burke–Ernzerhof (PBE) exchange-correlation functional^58^ within the generalized gradient approximation (GGA) and fully relativistic norm-conserving pseudopotentials generated using the optimized norm-conserving Vanderbilt pseudopotentials^59^ were used in the calculations. The primitive Brillouin zone was sampled by using a 10 × 10 × 10 Monkhorst–Pack *k* mesh, and a plane-wave energy cut-off of 900 eV was used. The Fermi surface calculation was performed on a dense *k* mesh of 41 × 41 × 41 and was visualized by using XCrysDen software^60^. The QE calculations were also verified using the projector-augmented wave (PAW)^61^ method as implemented in the Vienna ab initio simulation package (VASP)^62^ which gave similar results.

## Supplementary information

Supplementary Information

## Data Availability

The data generated in this study are provided in the Source Data file. Source data are provided with this paper.

## Source data

Source Data
