# Supplementary material for: Colossal Nernst power factor in topological semimetal NbSb2
Source: Nat Commun. 2022 Dec 9;13:7612. doi: 10.1038/s41467-022-35289-z (PMC9734562; doi:10.1038/s41467-022-35289-z)
Supplement: Supplementary file 1 — Supplementary Information [file 41467_2022_35289_MOESM1_ESM.pdf]

Supplementary Information

Colossal Nernst power factor in topological semimetal $NbSb_2$

Peng Li[1,2], Pengfei Qiu[1,2]*, Qing Xu[3], Jun Luo[1,2], Yifei Xiong[1,2], Jie Xiao[1], Niraj Aryal[4], Qiang Li[4,5], Lidong Chen[1,2], and Xun Shi[1,2]*

[1]State Key Laboratory of High Performance Ceramics and Superfine Microstructure, Shanghai Institute of Ceramics, Chinese Academy of Sciences, Shanghai 200050, China

[2]Center of Materials Science and Optoelectronics Engineering, University of Chinese Academy of Sciences, Beijing 100049, China

[3]Key Laboratory of Infrared Imaging Materials and Devices, Shanghai Institute of Technical Physics, Chinese Academy of Sciences, Shanghai 200083, China

[4]Condensed Matter Physics and Materials Science Division, Brookhaven National Laboratory, Upton, New York 11973-5000, USA

[5]Department of Physics and Astronomy, Stony Brook University, Stony Brook, New York 11794-3800, USA

**E-mail: qiupf@mail.sic.ac.cn; xshi@mail.sic.ac.cn*

Supplementary Note 1 Comparison of the DFT calculated and the quantum oscillation measured Fermi surface (FS) areas

In order to make comparison with the density functional theory (DFT) calculated and the quantum oscillation measured Fermi surface (FS) areas, we calculated the FS area of the extremal orbits on the *ab* plane for different values of the chemical potential μ. Supplementary Fig. 2 shows the dependence of the FS areas for the electron (blue squares) and hole (red circles) pockets as a function of μ. We find that the calculated FS areas are of similar order to the experimentally reported values[1] (227 T and 483 T) when μ is set to the value obtained from the DFT self-consistent calculation. This verifies that the calculated FS and band structure reported in the main text and

elsewhere in the literature, providing a reasonable description the experimental electronic structure. Note that unlike the electron pocket, the hole pocket gives multiple frequencies because of the corrugated FS topology along the *c*-direction. The quantum oscillation experiments may not always detect all the frequencies for a particular orientation of the magnetic field. To reveal all the extremal FS orbits, one needs to perform quantum oscillation measurements for different orientations of the magnetic field which, to the best of our knowledge, has not been performed in this system.

Supplementary Note 2 Influence of thermal Hall effect on the measured thermomagnetic properties

In this work, the Nernst thermopower, electrical conductivity, and thermal conductivity were measured under the adiabatic condition. During the measurement, the Righi-Leduc (thermal Hall) effect will produce a small temperature difference along the transverse direction, which might influence the measurement results. According to Scudder et al., the Nernst thermopower measured under the adiabatic condition (S_{yx}^{adi}) can be written as[2]

$$S_{yx}^{\mathrm{adi}} = S_{yx}^{\mathrm{iso}} + S_{yy}^{\mathrm{iso}} \frac{\nabla_y T}{\nabla_x T} \quad (1)$$

where the first term in the right of the equation represents the Nernst thermopower under the isothermal condition and the second term represents the contribution from the thermal Hall effect. The temperature gradient along the transverse direction ($\nabla_y T$) is generated by the temperature gradient along the longitudinal direction ($\nabla_x T$), thus $\nabla_y T/\nabla_x T$ is much less than 1. Likewise, considering the semimetal feature of $NbSb_2$, the S_{yy}^{iso} should be also much less than the S_{yx}^{iso}, which can be confirmed by the experiment results in this work that the S_{xx}^{adi} is orders of magnitude lower than the S_{yx}^{adi} under the same magnetic field below 100 K. Thus, it can be concluded that the thermal Hall effect has little influence on the Nernst thermopower, that is, $S_{yx}^{\mathrm{adi}} \approx S_{yx}^{\mathrm{iso}}$. We used the alternating current to measure the electrical conductivity, thus the thermal Hall effect has no influence on the accuracy of the measurement. Likewise, the

isothermal thermal conductivity (κ_{xx}^{iso}) and adiabatic thermal conductivity (κ_{xx}^{adi}) are related by[3]

$$\kappa_{xx}^{\text{iso}} = \frac{\kappa_{xx}^{\text{adi}}}{1+z_{\text{N}}^{\text{iso}}T} \tag{2}$$

The isothermal Nernst figure-of-merit ($z_{\text{N}}^{\text{iso}}T$) and adiabatic Nernst figure-of-merit ($z_{\text{N}}^{\text{adi}}T$) are related by[3]

$$z_{\text{N}}^{\text{iso}}T = \frac{z_{\text{N}}^{\text{adi}}T}{1-z_{\text{N}}^{\text{adi}}T} \tag{3}$$

Based on the κ_{xx}^{adi} and $z_{\text{N}}^{\text{adi}}T$, the κ_{xx}^{iso} and $z_{\text{N}}^{\text{iso}}T$ are calculated and shown in Supplementary Figs. 6a-b, respectively. No matter for thermal conductivity or figure-of-merit, the maximum difference between the adiabatic condition and isothermal condition is less than 16%, indicating the slight influence of thermal Hall effect.

Supplementary Note 3 Two-carrier model with constant relaxation time approximation

Under an external electrical field $\boldsymbol{E}$ and a temperature gradient ∇T, the charge current and thermal current can be expressed as

$$\begin{pmatrix} \boldsymbol{J_e} \\ \boldsymbol{J_q} \end{pmatrix} = \begin{pmatrix} \hat{\sigma} & \hat{\alpha} \\ \hat{\hat{\alpha}} & \hat{\kappa} \end{pmatrix} \begin{pmatrix} \boldsymbol{E} \\ -\nabla T \end{pmatrix} \tag{4}$$

where $\hat{\sigma}$ and $\hat{\kappa}$ are electrical conductivity tensor and thermal conductivity tensor, respectively. $\hat{\alpha}$ and $\hat{\hat{\alpha}}$ are thermoelectrical conductivity tensors with the relation[4] of $\hat{\hat{\alpha}} = \hat{\alpha}T$. Considering a two-dimensional case and $\boldsymbol{J_e} = 0$, we can get the Seebeck thermopower S_{xx} and Nernst thermopower S_{yx} as

$$S_{xx} = \frac{E_x}{\nabla_x T} = \frac{\sigma_{xx}\alpha_{xx}+\sigma_{yx}\alpha_{yx}}{\sigma_{xx}^2+\sigma_{yx}^2} \tag{5}$$

$$S_{yx} = \frac{E_y}{\nabla_x T} = \frac{\sigma_{xx}\alpha_{yx}-\sigma_{yx}\alpha_{xx}}{\sigma_{xx}^2+\sigma_{yx}^2} \tag{6}$$

When the electrical transports are simultaneously dominated by the electrons (e) and holes (h), that is, the two-carrier case,

$$\sigma_{xx} = \sigma_{xx}^{\text{e}} + \sigma_{xx}^{\text{h}} \tag{7}$$

$$\sigma_{yx} = \sigma_{yx}^{\text{e}} + \sigma_{yx}^{\text{h}} \tag{8}$$

$$\alpha_{xx} = \alpha_{xx}^{\mathrm{e}} + \alpha_{xx}^{\mathrm{h}} \quad (9)$$

$$\alpha_{yx} = \alpha_{yx}^{\mathrm{e}} + \alpha_{yx}^{\mathrm{h}} \quad (10)$$

According to the Drude model, when the applied magnetic field is along the *z* axis and perpendicular to the *xy* plane, the electrical tensor components can be expressed as

$$\sigma_{xx} = \frac{n_{\mathrm{e}} e \mu_{\mathrm{e}}}{1+\mu_{\mathrm{e}}^2 B^2} + \frac{n_{\mathrm{h}} e \mu_{\mathrm{h}}}{1+\mu_{\mathrm{h}}^2 B^2} \quad (11)$$

$$\sigma_{yx} = \frac{n_{\mathrm{e}} e \mu_{\mathrm{e}}^2 B}{1+\mu_{\mathrm{e}}^2 B^2} - \frac{n_{\mathrm{h}} e \mu_{\mathrm{h}}^2 B}{1+\mu_{\mathrm{h}}^2 B^2} \quad (12)$$

Therefore, the resistivity tensor components can be written as

$$\rho_{xx}(B) = \frac{\sigma_{xx}}{\sigma_{xx}^2+\sigma_{yx}^2} = \frac{1}{e}\frac{(n_{\mathrm{h}}\mu_{\mathrm{h}}+n_{\mathrm{e}}\mu_{\mathrm{e}})+(n_{\mathrm{h}}\mu_{\mathrm{e}}+n_{\mathrm{e}}\mu_{\mathrm{h}})\mu_{\mathrm{e}}\mu_{\mathrm{h}}B^2}{(n_{\mathrm{h}}\mu_{\mathrm{h}}+n_{\mathrm{e}}\mu_{\mathrm{e}})^2+(n_{\mathrm{h}}-n_{\mathrm{e}})^2\mu_{\mathrm{e}}^2\mu_{\mathrm{h}}^2B^2} \quad (13)$$

$$\rho_{yx}(B) = \frac{-\sigma_{yx}}{\sigma_{xx}^2+\sigma_{yx}^2} = \frac{B}{e}\frac{(n_{\mathrm{h}}\mu_{\mathrm{h}}^2-n_{\mathrm{e}}\mu_{\mathrm{e}}^2)+(n_{\mathrm{h}}-n_{\mathrm{e}})\mu_{\mathrm{e}}^2\mu_{\mathrm{h}}^2B^2}{(n_{\mathrm{h}}\mu_{\mathrm{h}}+n_{\mathrm{e}}\mu_{\mathrm{e}})^2+(n_{\mathrm{h}}-n_{\mathrm{e}})^2\mu_{\mathrm{e}}^2\mu_{\mathrm{h}}^2B^2} \quad (14)$$

According to Supplementary Equation (5), (6), (9), and (10), we convert thermoelectrical conductivity as the function of thermopower and electrical conductivity:

$$\alpha_{xx} = S_{xx}^{\mathrm{e}}\sigma_{xx}^{\mathrm{e}} - S_{yx}^{\mathrm{e}}\sigma_{yx}^{\mathrm{e}} + S_{xx}^{\mathrm{h}}\sigma_{xx}^{\mathrm{h}} - S_{yx}^{\mathrm{h}}\sigma_{yx}^{\mathrm{h}} \quad (15)$$

$$\alpha_{yx} = S_{xx}^{\mathrm{e}}\sigma_{yx}^{\mathrm{e}} + S_{yx}^{\mathrm{e}}\sigma_{xx}^{\mathrm{e}} + S_{xx}^{\mathrm{h}}\sigma_{yx}^{\mathrm{h}} + S_{yx}^{\mathrm{h}}\sigma_{xx}^{\mathrm{h}} \quad (16)$$

Substituting Supplementary Equation (7), (8), (15), and (16) into Supplementary Equation (5) and (6), we can get the S_{xx} and S_{yx} in the two-carrier model:

$$S_{xx} = [S_{xx}^{\mathrm{e}}(\sigma_{xx}^{\mathrm{e}}\sigma_{xx} + \sigma_{yx}^{\mathrm{e}}\sigma_{yx}) + S_{xx}^{\mathrm{h}}(\sigma_{xx}^{\mathrm{h}}\sigma_{xx} + \sigma_{yx}^{\mathrm{h}}\sigma_{yx}) + S_{yx}^{\mathrm{e}}(\sigma_{xx}^{\mathrm{e}}\sigma_{yx} - \sigma_{yx}^{\mathrm{e}}\sigma_{xx})$$
$$+S_{yx}^{\mathrm{h}}(\sigma_{xx}^{\mathrm{h}}\sigma_{yx} - \sigma_{yx}^{\mathrm{h}}\sigma_{xx})]/(\sigma_{xx}^2 + \sigma_{yx}^2) \quad (17)$$

$$S_{yx} = [S_{xx}^{\mathrm{e}}(\sigma_{yx}^{\mathrm{e}}\sigma_{xx} - \sigma_{xx}^{\mathrm{e}}\sigma_{yx}) + S_{xx}^{\mathrm{h}}(\sigma_{yx}^{\mathrm{h}}\sigma_{xx} - \sigma_{xx}^{\mathrm{h}}\sigma_{yx}) + S_{yx}^{\mathrm{e}}(\sigma_{xx}^{\mathrm{e}}\sigma_{xx} + \sigma_{yx}^{\mathrm{e}}\sigma_{yx})$$
$$+S_{yx}^{\mathrm{h}}(\sigma_{yx}^{\mathrm{h}}\sigma_{yx} + \sigma_{yx}^{\mathrm{h}}\sigma_{yx})]/(\sigma_{xx}^2 + \sigma_{yx}^2) \quad (18)$$

The S_{xx} and S_{yx} in single parabolic band model are given as[5]

$$S_{xx}^{i} = \frac{k_{\mathrm{B}}}{e}\left[\frac{\langle\tau_i E_i\rangle}{\langle\tau_i\rangle} - E_{\mathrm{F}}^{i}\right]\frac{1}{k_{\mathrm{B}}T} \quad (19)$$

$$S_{yx}^{i} = \frac{\mu_i B}{1+\mu_i^2 B^2}\frac{k_{\mathrm{B}}}{e}\left[\frac{\langle\tau_i^2 E_i\rangle}{\langle\tau_i^2\rangle} - \frac{\langle\tau_i E_i\rangle}{\langle\tau_i\rangle}\right]\frac{1}{k_{\mathrm{B}}T} \quad (20)$$

where *i* stands for electrons *e* or holes *h*, and $\langle\tau^p E^q\rangle = \int_0^\infty \frac{E^{q+3/2}\tau^p}{1+\mu^2B^2}\frac{\partial f_0}{\partial(-E)}dE$.

The energy overlap in $NbSb_2$ is 350 meV, which is much larger than $k_{\mathrm{B}}T$ (26 meV

at 300 K), so it can be regarded as a strong degenerate system below room temperature. In the strong degenerate system, the transport properties of the material are determined only by the scattering of charge carriers on the Fermi surface[6]. Therefore, the relaxation time can be approximated as a constant $\tau(E_{\mathrm{F}})$, where E_F is the Fermi level. Under the constant relaxation time approximation, the average energy of the carriers in the Hall current $\frac{\langle\tau_i^2 E_i\rangle}{\langle\tau_i^2\rangle}$ is equal to the average energy of the carriers in the drift current $\frac{\langle\tau_i E_i\rangle}{\langle\tau_i\rangle}$. According to Supplementary Equation (20), the S_{yx}^i in single band will be zero[7], which is called Sondheimer cancellation. When $\sigma_{yx}^2 \ll \sigma_{xx}^2$, the S_{xx} and S_{yx} in the two-carrier model with constant relaxation time approximation can be expressed as

$$S_{xx} = \frac{S_{xx}^{\mathrm{e}}(\sigma_{xx}^{\mathrm{e}}\sigma_{xx}+\sigma_{yx}^{\mathrm{e}}\sigma_{yx})+S_{xx}^{\mathrm{h}}(\sigma_{xx}^{\mathrm{h}}\sigma_{xx}+\sigma_{yx}^{\mathrm{h}}\sigma_{yx})}{\sigma_{xx}^2} \quad (21)$$

$$S_{yx} = \frac{S_{xx}^{\mathrm{e}}(\sigma_{yx}^{\mathrm{e}}\sigma_{xx}-\sigma_{xx}^{\mathrm{e}}\sigma_{yx})+S_{xx}^{\mathrm{h}}(\sigma_{yx}^{\mathrm{h}}\sigma_{xx}-\sigma_{xx}^{\mathrm{h}}\sigma_{yx})}{\sigma_{xx}^2} \quad (22)$$

For simplicity, Supplementary Equation (22) can be written as

$$S_{yx} = \frac{\sigma_{xx}^{\mathrm{e}}\sigma_{xx}^{\mathrm{h}}(\mu_{\mathrm{e}}+\mu_{\mathrm{h}})B}{(\sigma_{xx}^{\mathrm{e}}+\sigma_{xx}^{\mathrm{h}})^2}(S_{xx}^{\mathrm{h}} - S_{xx}^{\mathrm{e}}) \quad (23)$$

In the ideal case that $n_e = n_h$ and $\mu_e = \mu_h = \bar{\mu}$, the electrons and holes are completely compensated and the transverse built-in electric field is zero. Then, Supplementary Equation (23) can be rewritten as

$$S_{yx} = \frac{\bar{\mu}B}{2}(S_{xx}^{\mathrm{h}} - S_{xx}^{\mathrm{e}}) \quad (24)$$

Supplementary Note 4 Estimation of Seebeck thermopower (S_{xx}^i)

According to Supplementary Equation (24), the Nernst thermopower S_{yx} is in proportional to the $(S_{xx}^{\mathrm{h}} - S_{xx}^{\mathrm{e}})$, where S_{xx}^{e} and S_{xx}^{h} are the respective Seebeck thermopower of electrons and holes. By using Supplementary Equation (21) and (22), and the fitted n_e, n_h, μ_e, and μ_h, we can calculate the S_{xx}^i of single-crystalline $NbSb_2$ under different temperature and magnetic field. The results are plotted in Supplementary Fig. 9. Both S_{xx}^{e} and S_{xx}^{h} show weak dependence with the magnetic field, but strong dependence with the temperature.

Supplementary Note 5 Estimation of Seebeck thermopower caused by phonon-drag effect (S_p^i)

In the degenerate limit, the S_d^i related to the charge carrier diffusion processes is expressed as[8]

$$S_\mathrm{d}^i = \mp \frac{\pi^2\left(\lambda+\frac{3}{2}\right)k_B^2 T}{3eE_\mathrm{F}^i} \tag{25}$$

where λ is scattering factor and E_F^i is Fermi level. Above 100 K, the phonon-drag effect is negligible and S_{xx}^i roughly equals to S_d^i. Then, we can extrapolate the S_{xx}^i to 5 K based on the relationship of $S_\mathrm{d}^i \propto T$. The slopes are -0.215 $\mu VK^{-1}T^{-1}$ and 0.143 $\mu VK^{-1}T^{-1}$ for electrons and holes, respectively. Finally, the S_p^i below 100 K can be obtained[9] by subtracting the S_d^i from the S_{xx}^i. The results are shown in Fig. 4f.

Supplementary Note 6 Details about thermal conductivity and Nernst thermopower measurements by using four-probe method

The adiabatic thermal conductivity (κ_{xx}) and Nernst thermopower (S_{yx}) was simultaneously measured by using four-probe method on a modified thermal transport option (TTO) platform (Supplementary Fig. 11a). As shown in Supplementary Fig. 11b, we firstly used $Sn_{99}Bi_1$ alloy to fix the two thermometers on the $NbSb_2$ sample's surface. Then, the two ends of $NbSb_2$ sample were welded on the gold-plated copper sheets connecting to heat source/sink by using $Sn_{64}Bi_{35}Ag_1$ alloy. Finally, the copper wires for S_{yx} measurement were soldered symmetrically on the longitudinal sides of the sample by using In-Sn-Bi alloy. The κ_{xx} and S_{yx} was measured under positive and negative magnetic fields (B = ±1 T, ±3 T, ±5 T, ±7 T, and ±9 T). The S_{yx} data used for calculating the Nernst power factor and Nernst figure-of-merit are obtained by $S_{yx}=[S_{yx}(+B)-S_{yx}(-B)]/2$ to eliminate the effects of contact misalignments. In addition, via comparing with the κ_{xx} of the sample with and without adhering Cu wires (Supplementary Fig. 12), it is concluded that the Cu wires have little influence on the measurement.

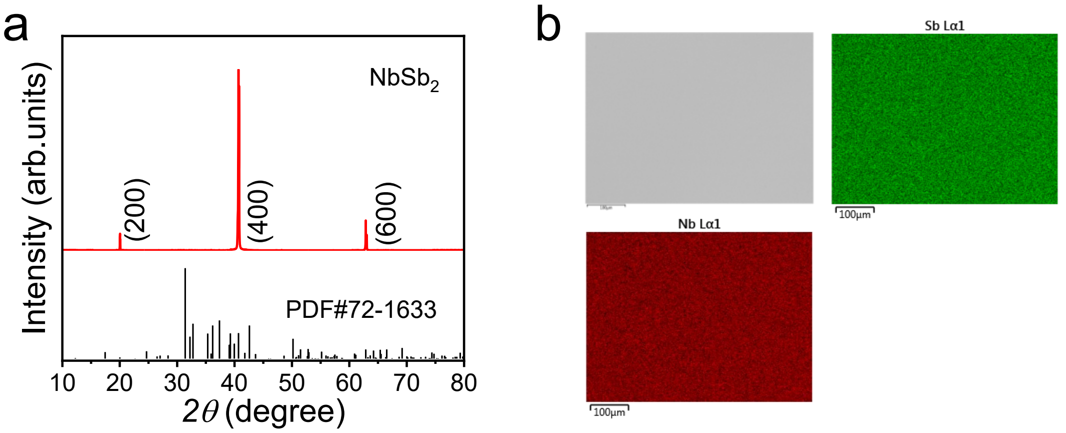

Supplementary Figure 1. **XRD and EDS measurements on $NbSb_2$ single crystal. a** X-ray diffraction pattern performed on the upper surface of $NbSb_2$ single crystal. **b** Backscattered electron (BSE) image and Energy dispersive X-ray spectroscopy (EDS) mapping performed on $NbSb_2$ single crystal.

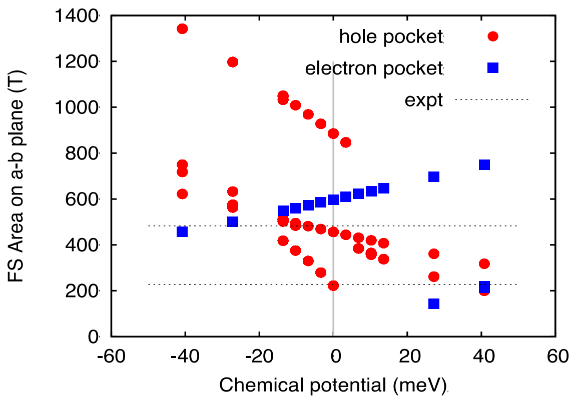

Supplementary Figure 2. **Variation of the calculated electron and hole Fermi surface cross-section areas as a function of the chemical potential μ.** $\mu = 0$ implies the Fermi level is placed at the DFT calculated value. The Fermi surface for this case is shown in the main text (Fig. 2d).

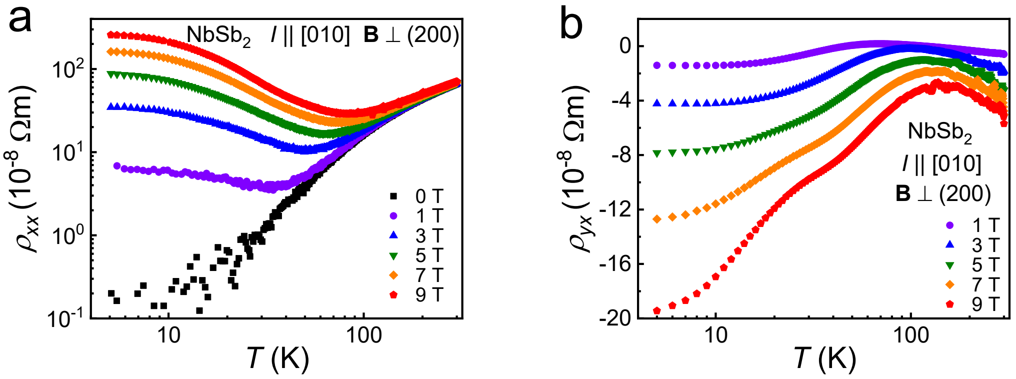

Supplementary Figure 3. **Electrical transport properties of single-crystalline $NbSb_2$ under different magnetic fields.** Temperature dependences of **a** transverse resistivity ρ_{xx} and **b** Hall resistivity ρ_{yx}.

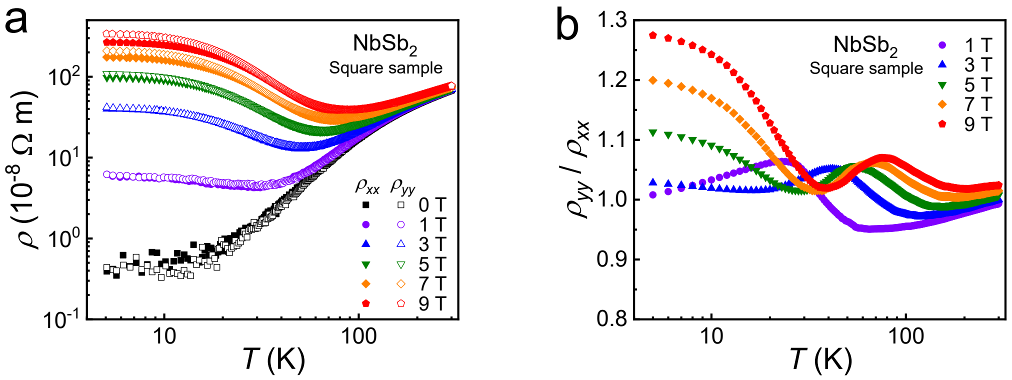

Supplementary Figure 4. **Isotropic electrical transport properties of single-crystalline $NbSb_2$ in the *bc* plane. a** Temperature dependence of electrical resistivities along *b*-axis (ρ_{xx}) and along *c*-axis (ρ_{yy}) measured by the four-probe method under various magnetic fields. The measurement was performed on a square sample with the dimension of 1.73×1.65×0.63 mm^3. **b** Temperature dependence of the ratio of longitudinal resistivity and transverse resistivity ρ_{yy}/ρ_{xx} under various magnetic fields. The maximum ρ_{yy}/ρ_{xx} is about 1.27 at 5 K under 9 T. It is decreased under lower magnetic field or at higher temperature. For example, the ρ_{yy}/ρ_{xx} is just about 1.02 at 300 K under 9 T and about 0.99 under 1 T.

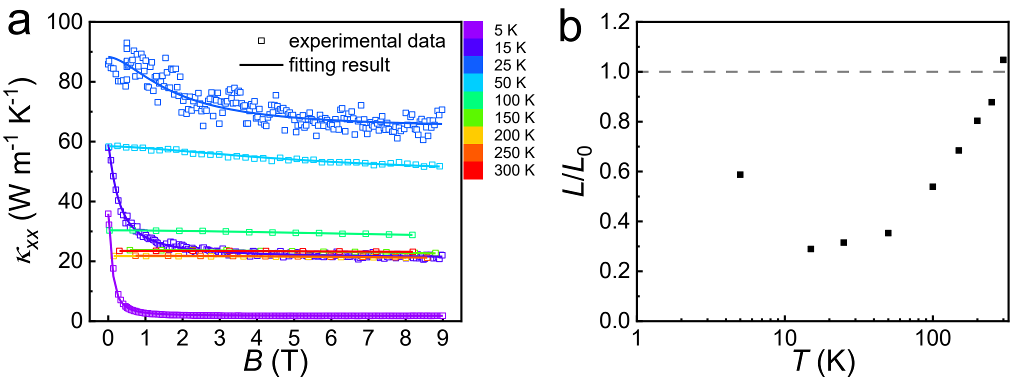

Supplementary Figure 5. **Violation of the Wiedemann-Franz law. a** Measured thermal conductivity κ_{xx} as a function of magnetic field B at different temperatures for single-crystalline $NbSb_2$. The symbols are experimental data and the lines are the fitting curves. **b** The ratio between Lorentz number L and the Sommerfeld value L_0 as a function of temperature at $B = 0$.

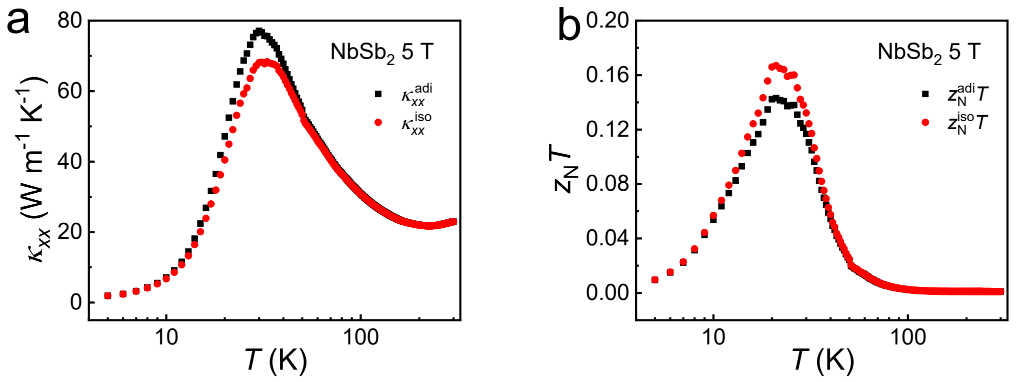

Supplementary Figure 6. **Comparisons on the results of adiabatic condition and isothermal condition. a** Adiabatic thermal conductivity $\kappa_{xx}^{\mathrm{adi}}$ and isothermal thermal conductivity $\kappa_{xx}^{\mathrm{iso}}$ for single-crystalline $NbSb_2$ under 5 T. **b** Adiabatic Nernst figure-of-merit $z_{\mathrm{N}}^{\mathrm{adi}}T$ and isothermal Nernst figure-of-merit $z_{\mathrm{N}}^{\mathrm{iso}}T$ for single-crystalline $NbSb_2$ under 5 T.

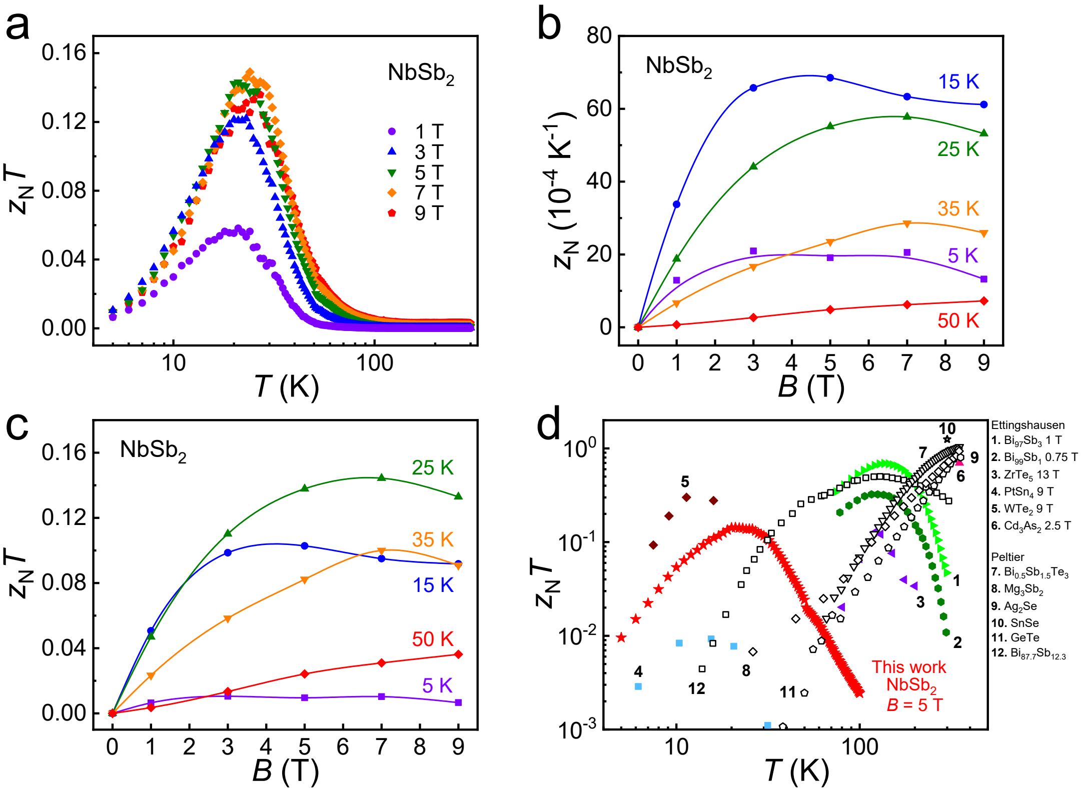

Supplementary Figure 7. **Nernst figure-of-merit (z_N and $z_N T$) for single-crystalline $NbSb_2$. a** Temperature dependence of $z_N T$ of single-crystalline $NbSb_2$ under different magnetic fields. Magnetic field dependence of **b** z_N and **c** $z_N T$ of single-crystalline $NbSb_2$ under different temperatures. **d** Comparison of $z_N T$ for single-crystalline $NbSb_2$ and other thermomagnetic materials[10-21].

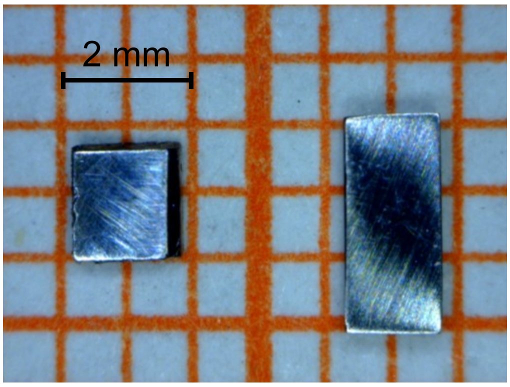

Supplementary Figure 8. **Optical images of the thin square and rectangle samples cut from the $NbSb_2$ single-crystal.**

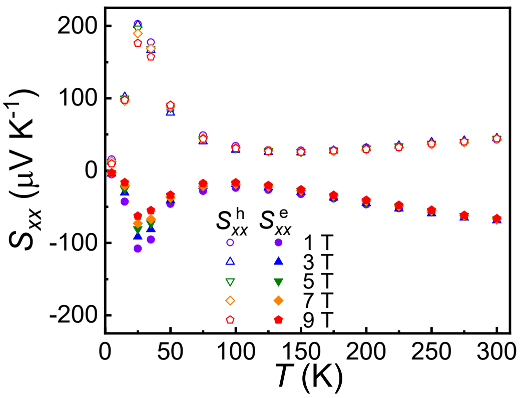

Supplementary Figure 9. **Respective Seebeck thermopower of electrons S_{xx}^{e} and holes S_{xx}^{h} under different magnetic fields for single-crystalline $NbSb_2$ derived from the two-carrier model.**

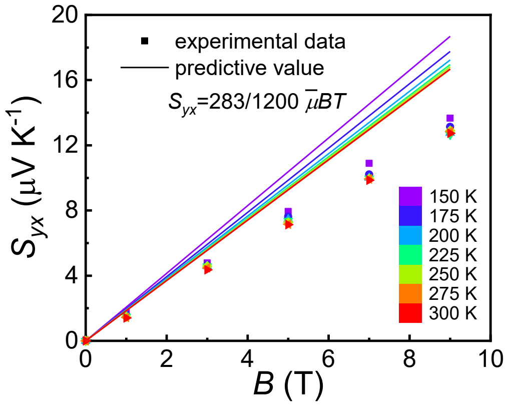

Supplementary Figure 10. **Comparison of experimental data and theoretical values of Nernst thermopower above 125 K.** In this temperature range, the carrier transports are dominated by the diffusion process. The predictive values are calculated by $S_{yx} = 283\bar{\mu}/E_{\mathrm{F}}T$ [22], where E_{F} = 1200 K is derived from the relation $E_{\mathrm{F}} = \frac{\hbar^2}{2m}(3\pi^2 n)^{2/3}$ [23], with the carrier concentration n equaling to 1.5×10^{20} cm^{-3} and m equaling to the electron mass m_0.

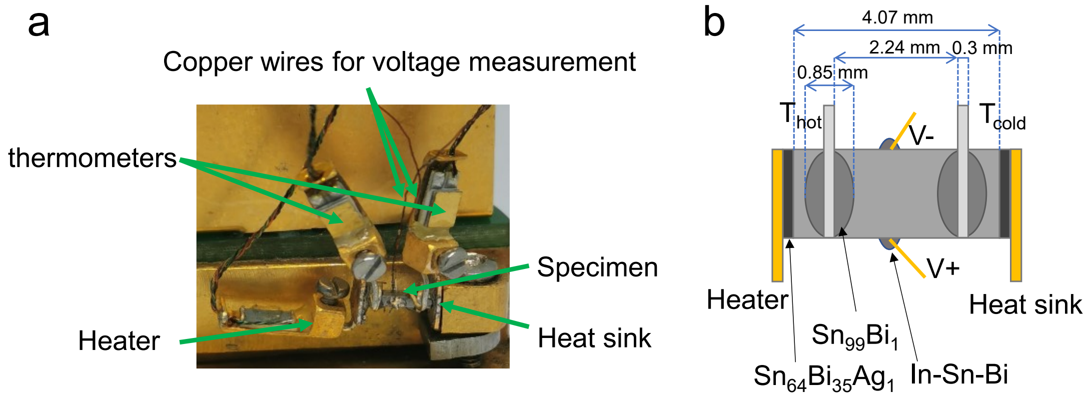

Supplementary Figure 11. **Thermal transport measurements of single-crystalline $NbSb_2$ a** Optical image for the sample and test platform. **b** Schematic diagram of the measurements of thermal conductivity κ and Nernst thermopower S_{yx} by using low melting-point alloys and four-probe method. Three kinds of low melting-point alloys, termed as $Sn_{99}Bi_1$, $Sn_{64}Bi_{35}Ag_1$, and In-Sn-Bi alloy, were chosen to fix the $NbSb_2$ sample on the test platform. The measurement error of thermal transports is within 20%.

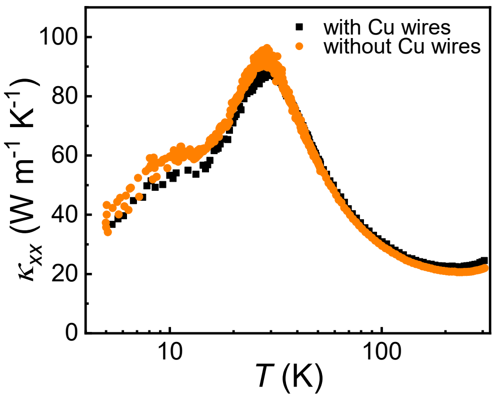

Supplementary Figure 12. **Thermal conductivity of single-crystalline $NbSb_2$ measured by using the four-probe method with and without the Cu wires adhering on the sample.**

Supplementary Table 1. **Parameters used to fit the measured thermal conductivity of single-crystalline $NbSb_2$.**

T (K)	κ_l (W m^{-1} K^{-1})	κ_e (0,T) (W m^{-1} K^{-1})	$\eta^{1/s}$ (T^{-1})	s
5	1.77	34.12	9.872	1.496
15	20.96	38.08	3.442	1.189
25	64.32	23.97	0.556	1.653
50	47.89	10.56	0.163	1.552
100	22.49	7.89	0.049	1.545
150	15.65	8.16	0.040	1.751
200	12.83	8.93	0.030	1.954
250	12.33	9.59	0.028	1.865
300	12.25	11.25	0.019	1.913

Supplementary references

1. Wang, K., Graf, D., Li, L., Wang, L. & Petrovic, C. Anisotropic giant magnetoresistance in $NbSb_2$. *Sci. Rep.* **4**, 7328 (2014).
2. Scudder, M. R., Koster, K. G., Heremans, J. P. & Goldberger, J. E. Adiabatic and isothermal configurations for Re_4Si_7 transverse thermoelectric power generators. *Applied Physics Reviews* **9**, 021420 (2022).
3. Delves, R. T. Figure of merit for Ettingshausen cooling. *Br. J. Appl. Phys.* **15**, 105-106 (1964).
4. Onsager, L. Reciprocal Relations in Irreversible Processes. I. *Phys. Rev.* **37**, 405-426 (1931).
5. Delves, R. T. Thermomagnetic effects in semiconductors and semimetals. *Rep. Prog. Phys.* **28**, 249-289 (1965).
6. Ye, L. *Semiconductor Physics (Second Edition) Part One*. (Higher Education Press, Beijing, 2007).
7. Ye, L. *Semiconductor Physics (Second Edition) Part Two*. (Higher Education Press, Beijing, 2009).
8. Blatt, F. J., Schroeder, P. A., Foiles, C. L. & Greig, D. *Thermoelectric power of metals*. (Plenum Press, New York and London, 1976).
9. Kagan, V. D., Red'ko, N. A., Rodionov, N. A., Pol'shin, V. I. & Zotova, O. V. Phonon drag thermopower in doped bismuth. *Physics of the Solid State* **46**, 1410-1419 (2004).
10. Cuff, K. F., Horst, R. B., Weaver, J. L., Hawkins, S. R., Kooi, C. F. & Enslow, G. M. The thermomagnetic figure of merit and Ettingshausen cooling in Bi–Sb alloys. *Appl. Phys. Lett.* **2**, 145-146 (1963).
11. Yim, W. M. & Amith, A. Bi-Sb alloys for magneto-thermoelectric and thermomagnetic cooling. *Solid-State Electron.* **15**, 1141-1165 (1972).
12. Xiang, J., et al. Large transverse thermoelectric figure of merit in a topological Dirac semimetal. *Sci. China: Phys., Mech. Astron.* **63**, 237011 (2019).
13. Wang, P., et al. Giant Nernst effect and field-enhanced transversal z_NT in $ZrTe_5$. *Phys. Rev. B* **103**, 045203 (2021).
14. Fu, C., et al. Largely suppressed magneto-thermal conductivity and enhanced magneto-thermoelectric properties in $PtSn_4$. *Research* **2020**, 4643507 (2020).
15. Lenoir, B., Cassart, M., Michenaud, J. P., Scherrer, H. & Scherrer, S. Transport properties of Bi-RICH Bi-Sb alloys. *J. Phys. Chem. Solids* **57**, 89-99 (1996).
16. Hao, F., et al. Roles of Cu in the Enhanced Thermoelectric Properties in $Bi_{0.5}Sb_{1.5}Te_3$. *Materials* **10**, 251 (2017).
17. Mao, J., et al. High thermoelectric cooling performance of n-type Mg_3Bi_2-based materials. *Science* **365**, 495-498 (2019).
18. Wang, L., et al. Discovery of low-temperature GeTe-based thermoelectric alloys with high performance competing with Bi_2Te_3. *J. Mater. Chem. A* **8**, 1660-1667 (2020).

19. Liang, J., et al. Crystalline Structure-Dependent Mechanical and Thermoelectric Performance in $Ag_2Se_{1-x}S_x$ System. *Research* **2020**, 6591981 (2020).
20. Qin, B., et al. Power generation and thermoelectric cooling enabled by momentum and energy multiband alignments. *Science* **373**, 556-561 (2021).
21. Pan, Y., He, B., Helm, T., Chen, D., Schnelle, W. & Felser, C. Ultrahigh transverse thermoelectric power factor in flexible Weyl semimetal WTe_2. *Nat. Commun.* **13**, 3909 (2022).
22. Behnia, K. & Aubin, H. Nernst effect in metals and superconductors: a review of concepts and experiments. *Rep. Prog. Phys.* **79**, 046502 (2016).
23. Gould, H. & Tobochnik, J. *Statistical and Thermal Physics with Computer Applications*. (Priceton University Press, Priceton. 2010)
